# Evaluation of a Public Health Intervention to Lower Mercury Exposure from Fish Consumption in Bermuda

**DOI:** 10.1371/journal.pone.0047388

**Published:** 2012-10-15

**Authors:** Eric Dewailly, Philippe Rouja, Martin Forde, Cheryl Peek-Ball, Suzanne Côté, Emma Smith, Olivia Drescher, Lyndon Robertson

**Affiliations:** 1 Department of Population Health and Environment, Research Center CHUQ, Québec, Canada; 2 Department of Conservation Services, Government of Bermuda, Flatts, Bermuda; 3 Department of Public Health and Preventive Medicine, School of Medicine, St. George’s University, St. George’s, Grenada, W.I; 4 Chief Medical Officer, Department of Health, Government of Bermuda, Hamilton, Bermuda; 5 Department of Biological and Chemical Sciences, University of the West Indies, Cave Hill, Barbados; 6 Windward Islands Research and Education Foundation (WINDREF), St. George’s University, St. George’s, Grenada, W.I; California Pacific Medicial Center Research Institute, United States of America

## Abstract

**Objectives:**

To assess the efficacy of a public health intervention to reduce blood mercury (Hg) concentration levels in pregnant Bermudian women.

**Methods:**

In 2003, we conducted a study entitled “Prenatal exposure of the Bermudian Population to Environmental Contaminants” which provided Bermuda’s first baseline data on prenatal exposure to several environmental contaminants, including Hg. The mean Hg concentration from 42 healthy newborns measured in umbilical cord blood was 41.3 nmol/L, ranging from 5–160 nmol/L. This concentration was much higher than expected, being approximately 8 times the general levels found in Canada and the U.S. Furthermore, we estimated that 85% of total Hg measured was in the form of methylmercury (MeHg), indicating that seafood consumption was the primary source of Hg exposure during pregnancy in Bermuda. Locally sourced seafood was identified as the most significant possible contributory source of Hg exposure. In 2005 the authors began a complementary research programme to study the levels of Hg in local commercial fish species. Coming out of this research were specific local fish consumption guidelines issued by the Department of Health advising pregnant women to avoid those local fish species found to be high in Hg while still encouraging consumption of fish species having lower Hg levels.

**Results:**

In 2010, under another research initiative, we returned to Bermuda to carry out another evaluation of Hg in human blood. Hg was measured in the blood of 49 pregnant women. The arithmetic mean Hg blood concentration was 6.6 nmol/L and the geometric mean 4.2 nmol/L. The maximum concentration found was 24 nmol/L.

**Conclusions:**

Hg exposure of Bermudian pregnant women has dropped significantly by a factor of around 5 since the foetal cord blood study in 2003.

## Introduction

Islanders such as Bermudians generally consume large quantities of fish. Most epidemiological and experimental studies on health related effects of exposure to mercury (Hg) strongly suggest that prenatal life is the most susceptible period for the induction of adverse effects on physical and neurological development [1], [2]. The consumption of certain types of fish has been associated with higher exposures to methylmercury (MeHg) [2]. Ecotoxicological studies also suggest that the problem of MeHg contamination is globalised and not restricted to industrialised countries but can be found in remote communities such as those in the Arctic [3]. Bermuda is the second most remote island community on the planet in the middle of the north Atlantic at 32 degrees latitude, some 600 nautical miles from the closest landmass.

### 2003 Evaluation of Prenatal Exposure of Bermudians to Hg

A study was conducted at the King Edward VII Memorial Hospital (KEMH) in Bermuda during the month of November2003 in order to provide baseline data on prenatal exposure to MeHg [4]. A total of (*n* = 42) women were recruited at the KEMH and written informed consent was obtained from all who agreed to participate in the study. At birth, a sample of the cord blood was collected and the delivering mother was asked to take a questionnaire which recorded basic information on potential sources of environmental contaminant exposures, such as diet and other daily lifestyle habits.

In this initial study, with the exception of one sample, all cord blood samples showed detectable levels of Hg, 25 out of 42 samples (59%) were above the U.S. EPA’s guideline for mercury in cord blood of 30 nmol/L (≈ 5.8 µg/L), and 3 out of 42 samples had cord blood mercury above the derived WHO guidelines of 90 nmol/L. Fortunately, no cord blood samples had Hg levels that exceeded the WHO’s lowest concentration Hg level associated with a 5% risk of developing psychomotor retardation of 200 nmol/L [1].

The arithmetic mean mercury content of cord blood was 41.3±4.7 nmol/L, with levels ranging from undetected to 160 nmol/L. For comparison, this mean level was a little more than 8 times the average concentration levels recorded in southern Quebec (*n* = 1109, geometric mean cord blood Hg concentration = 4.8 nmol/L, range: 1–67 nmol/L) [5].

Based on an analysis for inorganic mercury using two of the samples, it was estimated that 85% of total mercury measured in cord blood was methylmercury (MeHg), which strongly suggests that contaminated seafood was the main source of MeHg exposure. Hg exposure was associated with fish consumption based on the ratio of inorganic Hg to total Hg. Seafood available in Bermuda comes from a variety of sources with close to two-thirds being imported. An accompanying dietary seafood survey conducted with the mothers, however, suggested that local seafood might be an important contributory source of exposure. This was corroborated by a positive correlation found between the consumption of local fish and cord blood Hg levels. Data suggested that the consumption of two local fish species – wahoo and snapper, both predatory fish – were the main source of exposure to MeHg in Bermuda. This finding is important as it shows that local fish can be contaminated with significant levels of Hg, even in an apparently remote and pristine environment. Furthermore, these results suggest that such contamination is transferred to humans, including the foetus in the case of pregnant women, through consumption of certain fish species. Another key finding was that Hg cord blood levels were not associated with the consumption of other fish species, thus suggesting that appropriate guidelines could be created to limit the exposure to Hg while still maintaining the benefits to be gained from important nutrients found in fish.

### 2004 Evaluation of Hg Content in Fish Consumed by Bermudians

The sharing of these results with the Department of Health and Ministry of the Environment in 2004 resulted in the drafting of a specific advisory paper to the Cabinet. In view of the preliminary results linking local fish consumption to Hg in foetal cord blood, the Bermudian Government issued a request to the authors to conduct an in depth research programme to study the levels of Hg in local commercial fish species in order to better inform the general Bermuda population, particularly pregnant women [6].

A broad study of locally consumed fish (primarily commercial species) was started in 2004. Over 351 samples from 43 fish species were analysed. The investigation of Hg levels in Bermuda fish included an evaluation of two key beneficial nutrients; omega-3 fatty acids and selenium. High levels of omega-3 fatty acids from fish consumption have been linked to positive pregnancy outcomes in both the length of pregnancy (full term) and babies’ weight [7]. Since the goal of this study was not to undermine these positive aspects of fish consumption, the objective was to develop fish consumption advisories that balanced both the risk and the benefits of local fish consumption.

### 2007 Fish-consumption Guidelines for Bermudian Pregnant Women

The research was completed in 2007 and a report entitled “Balancing the Risks and Benefits of Local Fish Consumption” was presented to the Bermuda Cabinet and released to the public through a series of published articles in the most popular daily local newspaper. This research and resulting paper [6] was the basis for a set of specific local fish consumption guidelines issued by the Department of Health promoting fish with higher levels of n-3 fatty acids and advising pregnant women to avoid local fish species high in Hg; primarily the larger predator fish. In addition, all physicians received a copy of the final report and a detailed advisory letter from the Department of Health focusing on the counselling of patients on the relative risks and benefits of including local fish in their regular diets. Pregnant and nursing mothers and obstetricians were encouraged to use these guidelines to decide which local fish to consume and which to avoid during pregnancy.

### 2010 Evaluation Programme Reassessing Exposure of Bermudians to Hg

In 2010, through another Canadian funded research initiative, the Caribbean EcoHealth Programme (CEHP), we returned to Bermuda to conduct an evaluation of prenatal exposures to Persistent Organic Pollutant (POPs), heavy metals such as lead and mercury, and several pesticides. Given the earlier data collection effort, this study gave us the opportunity to assess if there was a change in Hg exposure since our first study in 2003 and since the issuing of specific recommendations for local fish consumption during pregnancy in 2007.

## Methods

This research project received approval from the government of Bermuda through the Bermuda Hospitals Board. The research project was also approved by Laval University’s ethical committee as well as the IRB of St. George’s University, Grenada. From March 23rd and November 26th 2010, 49 women were recruited at the Public Health Clinic and blood and urine were collected at their last prenatal visit prior delivery. Hg In maternal blood was measured by atomic absorption.

Results from Bermuda were compared with those from USA and Canada. The US data are extracted from the NHANES survey 2005–2006 [8]. The NHANES data set includes around 3000 thousand participants. In the case of Canada, comparative data were obtained from the Canadian Health Measure Survey (CHMS) which was conducted between 2007–2009 [9]. It is important to note that the CHMS samples were analysed at the National Institute of Public Health of Quebec (INSPQ) laboratory which was also the same laboratory used to do the analyses for this Bermuda study. In order facilitate the comparison of findings, we used the geometric means since the distribution of contaminants in human fluids is often log normal (skewed to the right). For the 14 samples where no Hg was detected (<2 nmol/L), half of the detection limit was assigned to these sample points.

## Results

The mean age of the 49 participating mothers was 23.4 yrs (IC 95% 21.9–24.9). The average birth weight was 3.183 gr (2.992–3.373). Hg was detected in 71% of mothers with a geometric mean of 4.2 nmol/L (arithmetic mean 6.6 nmol/L; IC 95% 4.9–8.3). None of the samples exceeded Health Canada’s provisional blood guidance value of 8 µg/L or 39.8 nmol/L, calculated based on the existing provisional Tolerable Daily Intake for children, pregnant women and women of childbearing age [10].

## Discussion

**Figure 1:**
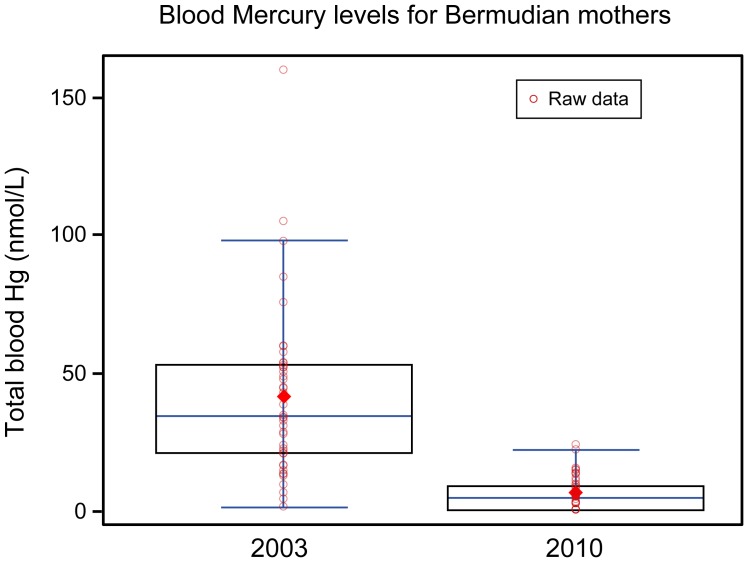
Total cord blood Hg levels (2003) and total blood mercury levels (2010) in Bermudian mothers illustrated with two boxplots showing the minimum (bottom whisker), first quartile, median, third quartile, maximum (top whisker) and the average (diamond). Raw data also shown in figure.

The arithmetic mean of Hg blood concentration in Bermudian mothers went from 41.3 nmol/L in 2003 to 6.6 nmol/L in 2010 illustrated in Figure 1. Even if we consider that cord blood Hg is generally higher (1.5 times) than the mothers’ blood Hg [11], this is still a very significant decrease in blood Hg concentrations levels by a factor of almost five. While it is true that the mean blood Hg concentration for Bermudian mothers (2010) may still seem high when compared to levels measured in low fish consumers, it is considerably lower than levels measured in populations which rely on the consumption of fish and sea-mammals for survival. While the mean blood Hg concentration for Bermudian mothers (2010) was twice as high as the most recent mean blood Hg reference level for Canadian females (n = 651) 20–39 years of age, measured by Health Canada (geometric mean: 3.5 nmol/L, 95% CI: 2.6–4.6 nmol/L) [9], the mean blood Hg concentration for Bermudian mothers was much lower than the level reported from a new study showing the effect of prenatal exposure to MeHg on brain function of Inuit children (mean cord Hg = 107.6 nmol/L, n = 269) [12].

In the first study (2003), women from all parishes entering the KEMH to deliver were asked to participate although in the second study (2010), participating pregnant women were only recruited at the public health clinic at their last prenatal visit. It is possible that in the second study lower social classes may have been overrepresented thus confounding comparisons of the two studies. However two things have happened in the interim between both studies. In 2003 most women who attended the public clinic were referred at their third trimester to a local obstetrician. Only two of the 46 were identifiable as clinic patients at time of birth, meaning that clinic and private patients were effectively blended for the 2003 study. In addition, due to the economic recession more women today are likely to use the clinic vs. private practice which would have broadened the socio economic pool in the 2010 study at the clinic. Furthermore the 2003 participants which were exclusively medically followed at the public health clinic did not differ in Hg concentration compared to the women seen in the private sector. And finally the spread of averages for Hg concentration in foetal cord blood in 2003 across all mothers was relatively homogenous and therefore does not suggest any potential groupings.

For the year 2003 there were 782 births in Bermuda, so the 42 births included in the 2003 study correspond to 65% of all the births which occurred during the month of November for that year. Therefore, although the number of participants is low, the sample is highly representative of the population.

As pointed out above two-thirds of fish consumed in Bermuda is imported. The study in 2003 suggested that the primary source of Hg exposure during pregnancy was local pelagic fish and analysis of local fish revealed some very high Hg levels in the larger pelagic fish. Although Bermuda’s fish eating habits have not been scientifically studied, there is ample anecdotal evidence to suggest that access to local fish specifically is not determined by socio economic class and may even be inversely related to socio economic class, with lower economic classes having more access to and consuming more local fish mainly due to grey market, recreational/subsistence type fishing, and cultural/traditional preferences by and of community members that favour local fish.

### Conclusions

In 2003, it was found that pregnant women in Bermuda were exposed to high levels of Hg. The consumption of predatory fish was identified as the major source of exposure and a complete assessment of Hg content of local fish species was undertaken. The findings of this assessment of local fish was disseminated and shared with the health authorities, local physicians and relayed by the media to the general public to avoid those fish species that had high levels of MeHg while encouraging the consumption of low Hg- high nutrient fish species. In 2010, a follow-up evaluation of blood Hg levels revealed a 4–5 fold decrease in average Hg levels. Further, none of the participant samples were found to exceed international guidelines. This intervention illustrates the positive effects that informed, precise, and well-targeted public health advisories, based on risk-benefit analyses, can have in helping to reduce exposure to Hg in fish-eating populations.
